# Molecular Mechanisms of the 1-Aminocyclopropane-1-Carboxylic Acid (ACC) Deaminase Producing *Trichoderma asperellum* MAP1 in Enhancing Wheat Tolerance to Waterlogging Stress

**DOI:** 10.3389/fpls.2020.614971

**Published:** 2021-01-18

**Authors:** Mamoona Rauf, Muhammad Awais, Aziz Ud-Din, Kazim Ali, Humaira Gul, Muhammad Mizanur Rahman, Muhammad Hamayun, Muhammad Arif

**Affiliations:** ^1^Department of Botany, Abdul Wali Khan University Mardan, Mardan, Pakistan; ^2^Graduate School of Biotechnology, College of Life Sciences, Kyung Hee University, Yongin-si, South Korea; ^3^Department of Biotechnology and Genetic Engineering, Hazara University Mansehra, Mansehra, Pakistan; ^4^National Agricultural Research Center (NARC), National Institute for Genomics and Advanced Biotechnology, Islamabad, Pakistan; ^5^Department of Biotechnology and Genetic Engineering, Islamic University, Kushtia, Bangladesh; ^6^Department of Biotechnology, Abdul Wali Khan University Mardan, Mardan, Pakistan

**Keywords:** ethylene, polyamines, waterlogging stress, wheat, *Trichoderma asperellum*, endophytic fungus, biofertilizer, ACC deaminase enzyme

## Abstract

Waterlogging stress (WS) induces ethylene (ET) and polyamine (spermine, putrescine, and spermidine) production in plants, but their reprogramming is a decisive element for determining the fate of the plant upon waterlogging-induced stress. WS can be challenged by exploring symbiotic microbes that improve the plant’s ability to grow better and resist WS. The present study deals with identification and application of 1-aminocyclopropane-1-carboxylic acid (ACC) deaminase-producing fungal endophyte *Trichoderma asperellum* (strain MAP1), isolated from the roots of *Canna indica* L., on wheat growth under WS. MAP1 positively affected wheat growth by secreting phytohormones/secondary metabolites, strengthening the plant’s antioxidant system and influencing the physiology through polyamine production and modulating gene expression. MAP1 inoculation promoted yield in comparison to non-endophyte inoculated waterlogged seedlings. Exogenously applied ethephon (ET synthesis inducer) and 1-aminocyclopropane carboxylic acid (ACC; ET precursor) showed a reduction in growth, compared to MAP1-inoculated waterlogged seedlings, while amino-oxyacetic acid (AOA; ET inhibitor) application reversed the negative effect imposed by ET and ACC, upon waterlogging treatment. A significant reduction in plant growth rate, chlorophyll content, and stomatal conductance was noticed, while H_2_O_2_, MDA production, and electrolyte leakage were increased in non-inoculated waterlogged seedlings. Moreover, in comparison to non-inoculated waterlogged wheat seedlings, MAP1-inoculated waterlogged wheat exhibited antioxidant–enzyme activities. In agreement with the physiological results, genes associated with the free polyamine (PA) biosynthesis were highly induced and PA content was abundant in MAP1-inoculated seedlings. Furthermore, ET biosynthesis/signaling gene expression was reduced upon MAP1 inoculation under WS. Briefly, MAP1 mitigated the adverse effect of WS in wheat, by reprogramming the PAs and ET biosynthesis, which leads to optimal stomatal conductance, increased photosynthesis, and membrane stability as well as reduced ET-induced leaf senescence.

## Introduction

Waterlogging stress (WS) reduces the photosynthesis rate and induces oxidative stress. It also accelerates leaf senescence and inhibits plant growth that leads to yield loss (Fukao et al., 2019; Park and Lee, 2019). The principal cause of damage of plants growing in waterlogged soil is due to an inadequate supply of oxygen to the submerged tissues (Colmer and Voesenek, 2009; Nishiuchi et al., 2012). Wheat is one of the major cereals in Asia and is rather waterlogging-sensitive, although flooding-tolerant relatives of *Triticum* have been reported such as *Triticum macha* L., *Triticum dicoccum* cv. *Pontus* (Davies and Hillman, 1988), and *Triticum spelta* (Burgos et al., 2001). However, *Triticum aestivum* L., a high-yielding and major cereal in Asia, is sensitive to waterlogged conditions. These conditions negatively affected shoot length, root/shoot dry weights, and leaf number of wheat seedlings (Yamauchi et al., 2018). The rich nutritional value of *T. aestivum* L. has increased the need to boost wheat production under WS conditions. Developing waterlogging tolerant varieties has long been a top priority for agriculturists using classical quantitative trait loci (QTL) approach and field trials but not much progress has been made in understanding the underlying molecular mechanism of waterlogging tolerance (WT) in plants. Moreover, studies at the transcriptomic, proteomic, and metabolomic level are required to understand the causes of wheat cultivars sensitivity to waterlogging and use these clues as a base for achieving flood-tolerant cultivars (Wang et al., 2016). An alternative promising approach has recently been exploited by using *Plant Growth Promoting Endophytic Fungus* (PGPEF) for quantification of flood tolerance in various plants. PGPEF is known to alleviate stress in plants by promoting ecological adaptability toward the harsh environment (Weyens et al., 2009; Khiralla et al., 2016). Positive plant–microbe associations have also been reported for WT in rice plants (Ferrando and Fernández Scavino, 2015). However, specific plant–fungal interactions for flooding tolerance have not been widely investigated in wheat, so far. Recently, endophytic *Aspergillus fumigatus* SG-17 isolated from *Myricaria laxiflora* (flood-tolerant plants) was reported to effectively alleviate flooding stress in *Arabidopsis thaliana*, by styrene antioxidant of (Z)-N-(4-hydroxystyryl) formamide (NFA) (Xue et al., 2020). *Trichoderma* species are ubiquitous, and some but not all *Trichoderma* strains are endophytes. Its inoculation in nutrient barren soil is known to enrich the soil quality by nutrient solubilization (Kapri and Tewari, 2010). *Trichoderma* exhibits biocontrol activity and also mitigates stress tolerance, including drought stress, resulting in plant resistance to pathogens through the systemic defense, thus enhancing plant growth with improved yield (Harman et al., 2004; Azarmi et al., 2011). The establishment of root colonization and chemical communication by *Trichoderma* strains has a strong impact on plant physiology by modifying plant gene expression for improvement in overall plant performance. Some strains of *Trichoderma* provide resistance not only to biotic stresses (diseases) but also to various abiotic stresses such as water deficits, salt, and temperature (Marra et al., 2006; Alfano et al., 2007; Djonović et al., 2007; Shoresh et al., 2010; Bae et al., 2011). *Trichoderma* strains have been used for induction of salt resistance in rice and drought resistance in wheat, rice, and tomato through metabolic changes (Rawat et al., 2012; Shukla et al., 2012; Pandey et al., 2016; Mona et al., 2017). Fungi including *Trichoderma* produce numerous secondary metabolites such as alkaloids, terpenoids, phenols, or flavonoids that enhance the activity of antioxidant enzymes or elicit a certain pathway for enhancing abiotic stress tolerance (McLellan et al., 2007; Mishra et al., 2016; Bilal et al., 2018). Likewise, several fungi have the considerable ability to produce certain enzymes such as 1-aminocyclopropane-1-carboxylate-deaminase (ACC deaminase) produced by *Trichoderma longibrachiatum* T6 (TL-6) involved in promoting wheat (*T. aestivum* L.) growth and enhancing plant tolerance to NaCl stress (Zhang et al., 2019). *T. asperellum* T203 produces ACC deaminase that regulates the endogenous ACC level and stimulates root elongation (Viterbo et al., 2010) and enhances plant tolerance to abiotic stress (Bal et al., 2013). It is known that ACC deaminase enzyme affects the ethylene (ET) levels when *Trichoderma* interacts with the plant roots. Presence of *ACC deaminase* gene in the genome of species of *Penicillium* and *Trichoderma* triggered the modulation in the production of ET by the fungal ACC deaminase and thus implicated in the plant tolerance response to several types of biotic and abiotic stresses (Harman et al., 2004). Fungal endophytes harboring ACC deaminase gene can cleave the ET precursor ACC to a-ketobutyrate and ammonia (Jia et al., 1999; Hontzeas et al., 2005; Yim et al., 2013). ET biosynthesis begins with S-adenosyl-L-methionine (S-AdoMet) and 1-aminocyclopropane-1-carboxylic acid (ACC) as precursors, and the main enzymes catalyzing this pathway are SAdoMet synthetase (SAM), ACC synthase (ACS), and ACC oxidase (ACO) (Wang et al., 2015). ET, the smallest phytohormone with the simple C_2_H_4_ structure is a gaseous phytohormone with roles in seed germination, fruit ripening, plant senescence, and plant immunity by affecting both the SA and the JA pathways (Bleecker and Kende, 2000; Pieterse et al., 2012). ET is produced in response to multiple environmental biotic and abiotic stresses, suggesting that it acts as a bridge between a changing environment and developmental adaptation. The multiple facets of ET as a signaling molecule have provided multiple molecular connections between ET and growth. The abiotic stress conditions that trigger ET synthesis include submergence, heat, shade, exposure to heavy metals and high salt, low nutrient availability, and water deficiency (Skirycz et al., 2011; Thao et al., 2015; Zhang et al., 2016; Dubois et al., 2017; Savada et al., 2017). The involvement of ET in flood-induced hyponastic leaf movement is already well known as the *A. thaliana aco5* mutants show reduced leaf hyponastic responses upon waterlogging (Rauf et al., 2013b). At the molecular level, transcriptomic changes have been observed in wheat roots interacted by *T. harzianum*, applied alone or in combination with different concentrations of calcium nitrate [Ca(NO_3_)_2_] as nitrogen (N) source. These changes revealed the role of *Trichoderma* as an inducer of plant defense (Rubio et al., 2019). *Trichoderma* spp. have also been reported as elicitors of wheat plant defense responses against *Septoria tritici* (Cordo et al., 2007). Several endophytic strains of *Trichoderma* including *T. afroharzianum*, *T. asperellum*, *T. longibrachiatum*, *T. lixii*, and *T. virens* have been known to increase plants’ photosynthetic capability (Harman et al., 2019). The effects of living *Trichoderma* on the plant transcriptome, metabolome, and proteome have been extensively studied (Marra et al., 2006; Chacón et al., 2007; Lorito et al., 2010; Morán-Diez et al., 2012; Mendoza-Mendoza et al., 2018). However, the role of *Trichoderma* in the interaction with the plant for WS tolerance has not yet been studied, specifically through bio-reduction of ET in the plant. The aim of the present study is to enhance WS tolerance by exploiting *Trichoderma* and identify the molecular base inducing WS tolerance response in wheat.

## Materials and Methods

### Isolation of Endophytic Fungi

For isolation of endophytic fungi, the *C. indica* L. plants were collected from the marshy lands near Tarbela lake, about 30 km from the city of Swabi (33°/55′ and 34°/23′ North-latitudes, 72°/13′ and 72°/49′ East-longitudes), built on the Indus river of Pakistan. Detached rhizome segments were surface sterilized with ethanol (70% for 2 min) and perchloric acid (1% for 30 s). After sterilization, final washing was done using double-distilled H_2_O to remove residues of perchloric acid and ethanol. To isolate endophytic fungus, the outer rough surface was removed, the inner part of the sterilized rhizome was obtained, and five segments per plate were placed on plates of autoclaved Hagem minimal medium (0.5% glucose, 0.05% KH_2_PO_4_, 0.05% MgSO_4_⋅7H_2_O, 0.05% NH_4_Cl, 0.1% FeCl_3_, 80 ppm streptomycin, and 1.5% agar; pH 5.6 ± 0.2). The emerged fungal colonies were separated and cultured on potato dextrose agar (PDA) medium plates, incubated at 25°C for 1 week. Fungal biomass and culture filtrates were collected by culturing pure colony in 50 ml of Czapek culture broth media (1% glucose, 1% peptone, 0.05% KCl, 0.05% MgSO_4_⋅7H_2_O, and 0.001% FeSO_4_⋅7H_2_O; pH 7.3 ± 0.2) and incubated at 30°C temperature, 120 rpm shaker for 7 days. Pellet of biomass was separated from the supernatant, lyophilized (ISE Bondiro Freeze Dryer), and stored at −70°C for further analysis, after harvesting the culture filtrates (CF) by centrifugation (4,000 × *g*, 4°C, and 15 min). Fungal culture filtrate was used for further analysis.

### PCR Amplification and Sequencing for MAP1 Identification

DNA was extracted from the selected strain by using the DNeasy plant mini kit (QIAGEN, Valencia, CA, United States), according to the method of Zhang et al. (2010). The selected endophytic fungus was identified by sequencing the internal transcribed regions (ITS) of 18S rDNA. The universal primers ITS1 (5-TCCG TAGGTGAACCTGCGG-3′) and ITS4 (5′-GCTGCGTTCTTCATCGATGC-3′) were used to amplify the ITS1-5.8S-ITS2 region of 18S rDNA (White et al., 1990).

### Characterization of the Fungal Endophyte MAP1

#### Quantification of ACC Deaminase Enzymatic Activity

The quantitative assessment of ACC deaminase enzymatic activity of fresh fungal culture was done spectrophotometrically in terms of α-ketobutyrate production at 540 nm using a method developed by Yedidia et al. (1999). Ketobutyrate (10–200 μmol) was used for the standard curve. To this end, 1 ml of spore suspension of MAP1 (1 × 10^8^ spores ml^–1^) was inoculated in 60 ml of synthetic medium supplemented with 0.5, 1.0, 1.5, and 2.0 mM of ACC (no ACC added in the control). The experimental procedure was adapted from the protocol of Zhang et al. (2019).

#### RNA Extraction and Gene Expression Analysis of *TasACCD* by RT-qPCR

To performe Real Time-quantitative PCR (RT-qPCR) total RNA was extracted according to the method of Viterbo et al. (2010). DNase-treated RNA was further cleaned using RNeasy Mini columns (Qiagen, Hilden, Germany), and the equal amount of RNA (2 μg) was converted to first standard cDNA using SuperScript II reverse transcriptase (Invitrogen, Lyon, France) according to the manufacturer’s procedure using oligo (dT) as a primer. Primer list is mentioned in Table 1. PCR reactions were run on an ABI PRISM 7900HT sequence detection system (Applied Biosystems Applera, Darmstadt, Germany), and amplification products were visualized using SYBR Green (Applied Biosystems Applera, Darmstadt, Germany). *Trichoderma* β*-tubulin* (AY390326) served as internal control previously reported by Viterbo et al. (2004).

**TABLE 1:** Primers used for RT-qPCR analysis.

Gene accession	Gene identifier	Primer sequences
***T. asperellum* primers for RT-qPCR**		
*FJ751936.1*	*ACC deaminase (TasACCD)*	*F-CGTCCAATCAAACCATACCC*
		*R-GCCGAAGAGTGATGGGTCTA*
*AY390326*	*Trichoderma b-tubulin*	*F-GACCTGCTCCACCATCTTCC*
		*R-CAGTGGAGTTGCCGACAAAG*
***T. aestivum* primers for RT-qPCR**		
*AB181991*	*TaActin*	*F-ACCTTCAGTTGCCCAGCAAT*
		*R-CAGAGTCGAGCACAATACCAGTTG*
*AY114121.1*	*T. asetivum calcium ion channel*	*F-CCCTCGTTGTGCTTTAATGG*
		*R-CACGGCTGCTACATACTCCA*
*KM199269.1*	*Guard cell S-type anion channel (TaSLAC1)*	*F-TCTCCGTTTCTCATGGGTTC*
		*R-TGGTGTTCCTTTTCCTTTGG*
*EU177559.1*	*Aquaporin (TaPIP2-6)*	*F-TGTCTCCGATGAACCCTTTC*
		*R-AGTCGGCACCAAGCATTTAC*
*JN129258.1*	*Heat Shock Protein 70 (TaHSP70)*	*F-TCCGTCATGGAAGGAAAGAC*
		*R-GGACCCACGAACTGTGTTCT*
*CV065593.1*	*Eukaryotic translation initiation factor 5A-1 (TaTIF5A-1)*	*F-CGTTCCTTCATCCCACAACT*
		*R-ATCTGGCCAAGCAGAACATC*
*X76532.1*	*Protochlorophyilide reductase (TaPORA)*	*F-CACCTTCTCGTCGCTCTACC*
		*R-CCTCCTGTGAGAGCTGGTTC*
*EF105406.1*	*Golden 2-like protein (TaGlk1)*	*F-CCACAAGTCGTCAAGCAAGA*
		*R-GTGCCGAGTGAGTGAGTTGA*
*AF251264.1*	*Ribulose bisphosphate carboxylase activase B (TaRcaB)*	*F-TCAAGGTCCCTCTCATCCTG*
		*R-GTTGATGAAGAGGCAGCACA*
*KX037456.1*	*ATP-dependent zinc metalloprotease (TaFTSH2)*	*F-AACACCTGGGTTCAGTGGAG*
		*R-ATGCCCAACTTCGTGGTAAG*
*MT521008.1*	*Ethylene responsive factor 8 (TaERF8-2A)*	*F-CAATATCGAGCTCAGGCACA*
		*R-TGCTAAGACATGTCCCGATG*
*KF900072.1*	*ACC oxidase (TaACO)*	*F-CTGCTAATGCTGCTGTCTCG*
		*R-CGCGAACTCCTTGAACTTGT*
*U42336.1*	*ACC synthase (TaACS2)*	*F-CGTGCGTACATTTGATTTGG*
		*R-ATTGCAACCTCCGTAAAACG*
*HM770452.1*	*Spermine synthase 2 (TaSPMS)*	*F-TGTGGCTGCACACACATCTA*
		*R-GGGTTCACAGGAGTCAGGAA*
*KF900082.1*	*Spermidine synthase (TaSPDS)*	*F-CCGACTTCATGTTGGTGATG*
		*R-TGCAGCCACATACTTTCAGC*
*EU236151.1*	*Arginine decarboxylase (TaADC)*	*F-ACTACCTGGGCATGTTCCTG*
		*R-CTTGAGCACCTCGAACATCA*
*HQ121401.1*	*S-adenosyl methionine decarboxylase (TaSAMDC)*	*F-GCGTCCTCATCTACCAGAGC*
		*R-CAGATGGAATAGCGACAGCA*

#### Analysis of the Endophytic Fungal Culture Filtrate

Salkowski reagent was used for IAA quantification in fungus by using the method of Benizri et al. (1998). To prepare the Salkowski reagent, 1 ml of 0.5 M FeCl_3_ was mixed with 50 ml of 35% HClO_4_. Two milliliters of Salkowski reagent was added to 1 ml of fungal culture filtrate (FCF) in test tubes and incubated for 30 min in the dark at room temperature. Salkowski reagent (4 ml) was used as control. Absorbance was measured at 540 nm using a UV/VIZ spectrophotometer (PerkinElmer Inc. United States). Proline content was determined in fungal culture filtrate using the procedure of Bates et al. (1973). Two milliliters of FCF was taken in separate 10 ml test tubes. Two milliliters of acid ninhydrin reagent (6 M of 20 ml of H_3_PO_4_, 30 ml of CH_3_COOH, and 1.25 g of ninhydrin) was added in each sample. The mixture was warmed in the water bath for 60 min. After cooling, 4 ml of toluene was added in each sample. Toluene layer was then separated with a dropper. Four milliliters of toluene was also used as blank. Absorption was taken at 520 nm using a UV/Vis spectrophotometer. Flavonoids have been determined in samples using the method of Khatiwora et al. (2012). Five milliliters of FCF was centrifuged for 15 min (10,000 rpm, 25°C) after addition of 5 ml of ethanol (80%), and 0.5 ml of extract was taken in separate 10 ml test tubes; 0.1 ml of potassium acetate, 0.1 ml of AlCl_3_ (10%), and 4.3 ml of 80% methanol were added in sequence and were shaken vigorously. After 30 min of incubation, absorbance was recorded at 415 nm (PerkinElmer Lambda 25 double beam spectrophotometer). Total phenols in culture filtrate were quantified by using the same method described by Khatiwora et al. (2012). Two milliliters of centrifuged FCF samples was taken in a 10 ml glass tube with 3 ml of distilled water; 0.5 ml folin-ciocalteau reagent (Sigma-Aldrich, Deisenhofen, Germany) was added (1:1 with water and 2 ml of 20% Na_2_CO_3_, sequentially in each tube). A blue color was formed in each tube because the phenols go through a complex redox reaction with phosphomolibidic acid in alkaline folin-ciocalteau reagent, resulting in a molybdenum blue complex. The test solutions were heated for 1 min and cooled, and absorption was taken at 650 nm. Folin reagent was used as a control. Chemicals and reagents were purchased from Sigma-Aldrich (Deisenhofen, Germany), Fluka (Buchs, Switzerland) and Merck (Darmstadt, Germany).

#### Colonization Frequency

The colonization frequency (% CF) of selected endophytic fungus was calculated according to Hata and Futai (1995) as:% CF = (Ncol/Nt)/100, where Ncol = number of segments colonized by each fungus and Nt = total number of segments studied.

#### Deaminase-Producing Fungus Screening by *eto1* Bioassay

ET-induced morphological modifications occurring during the growth of the etiolated *Arabidopsis* seedlings are easily detected by visual inspection. To assess the growth-stimulating and ET-biosynthesis inhibiting potential of MAP1, the hypocotyl growth kinetics were calculated for etiolated *eto1* mutant (Col-0 background) seedlings (Wang et al., 2004) with and without the application of culture filtrate. The *eto1* mutant and Col-0 seeds were surface-sterilized for 1 min in 70% ethanol and then in sterilization solution (20% sodium hypochlorite) for 30 min. After imbibition, the seeds were stratified at 4°C for 4 days before germination. Stratified seeds were sown on half-strength, liquid MS medium (Murashige and Skoog, 1962) supplemented with 1% (w/v) sucrose and growth in the dark at 23°C for 66 to 72 h on filter papers in the glass Petri plates.

There are four treatments in this experiment:

(1) Col-0 = −MAP1 (2 ml of MS liquid media + 2 ml of Czapek media alone without fungal culture)
(2) Col-0 = + MAP1 (2 ml of MS liquid media + 2 ml of Czapek media with fungal culture)
(3) *eto1* = −MAP1 (2 ml of MS liquid media + 2 ml of Czapek media alone without fungal culture)
(4) *eto1* = + MAP1 (2 ml of MS liquid media + 2 ml of Czapek media with fungal culture)

Plant–fungal co-culturing assay was performed by using three independent biological replicates with at least 15 seedlings per Petri plate. At least five Petri plates were prepared for each treatment.

### Wheat Seedling Assay

*T. aestivum* L. seeds were surface-sterilized for 1 min in 70% ethanol and then in sterilization solution (20% sodium hypochlorite) for 30 min. After imbibition, the seeds were stratified at 4°C for 3 days before germination. Stratified seeds were placed on wet filter papers in the Petri plates and four uniformly germinated seedlings were transferred in pots containing sterilized garden soil. Three grams of MAP1 mycelium was thoroughly pre-mixed to the sterilized garden soil. Likewise, 1 ml of MAP1 culture filtrate was applied onto the base of 7-day-old uniformly grown wheat seedlings, and pots without active culture biomass and culture filtrate were assigned as control. To this end, MAP1 biomass and culture filtrate inoculation to wheat was performed by using freshly prepared 50 ml of Czapek media of MAP1 culture. The wheat seedling assay was performed in triplicate, and each replicate is composed of 10 pots (6 × 8 cm in diameter and height) with four seedlings (total = 4 × 10 × 3 = 120 seedlings per treatment). Four milliliters of water was irrigated to each pot throughout the experiment. All the pots were kept at 25–28/15–17°C temperature (day/night), 300 μmol m^–2^ s^–1^ light intensity, 17/7 h photoperiod, and 70/85% relative humidity. To test the effect of ET biosynthesis on the waterlogging-induced stress response, 15-day-old seedlings were irrigated (8 h before the onset of flooding treatment) with 2 ml of 0.1 mM ACC (ET precursor) and 2 ml of 0.1 mM 2-chloroethyl phosphonic acid (ethephon; an ET producer). ACC and ethephon were prepared in an aqueous solution containing 0.1% Tween 20, while 2 ml of the aqueous solution containing 0.1% Tween 20 was irrigated in control plants. Similarly, to observe the counter effect of ET producers, 2 ml of 0.1 mM amino-oxyacetic acid (AOA; ET inhibitor) was irrigated to 15-day-old seedlings. All chemicals were purchased from Sigma-Aldrich (Deisenhofen, Germany). Eight hours after chemical supplementation, 15-day-old plants were exposed to waterlogging (WL) stress for 5 days continuously and were subjected to a recovery period of further 5 days. WS treatment was induced by immersing the pots (height 8 cm) in the plastic tanks (40:20:8 cm, length:width:height) and were exposed to flooding with tap water by filling up to 7.8 cm such that the upper water level always remained below the pot upper edge. For control treatments, plants were put inside the tanks without flooding.

There are seven treatments in this experiment:

(1) Control = no waterlogging stress
(2) WL = waterlogging stress
(3) WL + ET = waterlogging stress and ethephon (100 μM)
(4) WL + ACC = waterlogging stress and ACC (100 μM)
(5) WL + AOA = waterlogging stress and AOA (100 μM)
(6) MAP1 = endophyte inoculated
(7) WL + MAP1 = waterlogging stress and endophyte inoculated

Plants were sampled at three main time points:

(i) 0 day of flooding (onset of WS at 15 DAG)
(ii) 5 days after flooding (20 DAG)
(iii) 5 days after the recovery period (25 DAG)

### Morphological and Physiological Analysis of Plants

After harvesting the seedlings, different growth parameters including shoot length, root length, and fresh weight were recorded. Each treatment was replicated three times and the experiment was repeated three times.

#### Total Chlorophyll Content

Chlorophyll content of leaf was quantified by using a chlorophyll meter (SPAD-502, Minolta, Corp., Spectrum Technologies).

#### Growth Rate of Plants

The plant growth rate was evaluated by measuring the total shoot height before and after WS.

#### Stomatal Conductance (*g*_*s*_)

Before and after WL treatment, the *g*_*s*_ of the abaxial surface of leaf was measured by a leaf porometer (Decagon Devices, Inc., Pullman, WA, United States) at 10:00 a.m.

#### Visualization and Quantification for H_2_O_2_

H_2_O_2_ production was monitored by the histochemical staining method by using 3,3-diaminobenzidine (DAB), according to Thordal-Christensen et al. (1997). To this end, the H_2_O_2_ staining agent, DAB (Sigma-Aldrich, Deisenhofen, Germany), was dissolved in water and adjusted to pH 3.8 with KOH. To avoid auto-oxidation, the DAB solution was freshly prepared (Fryer et al., 2002). Segments (1.0 cm in length) of the 3rd leaf from 25-day-old plants were infiltrated under vacuum with 0.5 mg ml^–1^ DAB staining solution and further incubated for 12 h; chlorophyll was removed by incubating seedlings in 90% ethanol at 70°C for 10 min. H_2_O_2_ was visualized as brown color due to DAB polymerization. Leaf segment images were then captured with a digital camera (Canon). For quantification of H_2_O_2_ samples from the leaf, tissues were ground in liquid nitrogen, and 30 mg of ground frozen tissue from each sample was placed in an Eppendorf tube and kept frozen. One milliliter of 20 mM sodium phosphate buffer, pH 6.5, was immediately added into the tube and mixed. The extraction was centrifuged at 10,000 × *g* for 10 min at 4°C, and the supernatant was used for the assay. The quantification of H_2_O_2_ was assayed by monitoring the absorbance at 410 nm as reported previously (Moloi and van der Westhuizen, 2006).

#### Extraction of Antioxidant Enzymes

Wheat plants (25 days old) were harvested for determining enzymatic activities by using a spectrophotometer. Reduced glutathione (GSH) activity was quantified by measuring the oxidation of NADPH according to the method of Keutgen and Pawelzik (2008). Peroxidase (POD) activity was quantified following the protocol of Gorin and Heidema (1976). The activity of ascorbate peroxidase (APX) was calculated through ascorbic acid oxidation by the method of Asada (1992). Catalase (CAT) activity was calculated by following the method of Chandlee and Scandalios (1984). 2,2-Diphenyl-1-picrylhydrazyl (DPPH) free radical scavenging activity was calculated according to Chrysargyris et al. (2016).

#### Determination of Malondialdehyde (MDA) Content and Electrolyte Leakage

MDA content in 25-day-old wheat seedlings was measurement by using the method of Heath and Packer (1968). Electrolyte leakage was calculated as (Ci/Cmax) × 100.

#### Quantification of ACC Content and Free Polyamines

ACC contents in the roots of 25-day-old plants were quantified by using the method from Yu et al. (2016), and free polyamines were calculated in the leaf tissue of 25-day-old wheat seedlings as mentioned by Duan et al. (2008).

### Gene Expression Analysis by RT-qPCR in Wheat

Total RNA was extracted from 25-day-old wheat seedlings using the GeneJET Plant RNA Purification Kit (Thermo Scientific^TM^). Approximately 2 μg of total DNase-treated-RNA was reverse transcribed using the RevertAid First Strand cDNA Synthesis Kit by Invitrogen (Karlsruhe, Germany). Expression analysis of ET and polyamine biosynthesis genes in wheat was performed by using primers (Table 1), designed through primer 3.0 (Untergasser et al., 2012). *TaACTIN2* (*AB181991*) served as an internal control, previously reported by Xu et al. (2013). Amplification of each gene was performed in triplicate by using an ABI PRISM 7900HT sequence detection system (Applied Biosystems), and amplification products were visualized using SYBR Green (Applied Biosystems). Amplification curves were analyzed with a normalized reporter (Rn: the ratio of the fluorescence emission intensity of SYBR Green to the fluorescence signal of the passive reference dye). RT-qPCR expression analysis was performed by using three independent biological replicates with at least three technical replicates.

### Statistical Analysis

All phenotypic measurements and quantitative analysis were performed by using at least three independent biological replicates with at least five technical replicates. Analysis of variance (ANOVA) was used to analyze the data and DMRT (Duncan Multiple Range Test) at *p* < 0.05, via SPSS-20 (SPSS Inch., Chicago, IL, United States), was used to equate the mean of all values.

## Results

### Isolation of Endophytic Fungi

**FIGURE 1:**
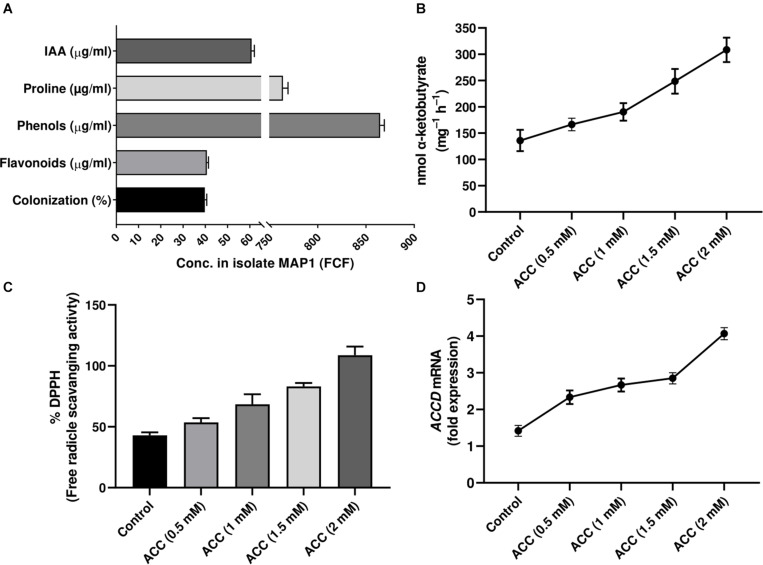
Characterization of MAP1 isolate. **(A)** IAA, proline, phenols, flavonoid content, and root colonization. **(B)** ACC deaminase activity. **(C)** %DPPH, free radical scavenging activity. **(D)** *ACC deaminase* gene expression of MAP1. Quantitative data represent means ± *SD* of three independent experiments and at least six technical replicates.

MAP1 was isolated from the rhizome of *C. indica* L. The culture filtrate (CF) of MAP1 grown in Czapek media had the highest amount of IAA, i.e., 60 ± 1.7 μg ml^–1^. MAP1 ably produced greater content of proline, phenols, and flavonoids (755 ± 1.3 μg ml^–1^, 863 ± 1.5 μg ml^–1^, and 40 ± 1.0 μg ml^–1^, respectively, Figure 1A).

### ACC Deaminase Activity by MAP1

MAP1 showed growth and potential for ACC deaminase activity on ACC supplemented Czapek media. ACC deaminase activity of MAP1 was noticed up to 140 nmol of α-ketobutyrate mg^–1^ h^–1^, which was further increased up to 330 nmol of α-ketobutyrate mg^–1^ h^–1^ (Figure 1B). Culture filtrate also exhibited significantly (*p* < 0.002) high DPPH free radical scavenging activity upon supplementation of ACC (Figure 1C).

### RT-qPCR for *TasACCD* Transcript Abundance

RT-qPCR analysis was performed for evaluating the transcript abundance and the data revealed an upregulation of *TasACCD* transcript in an ACC-dependent manner (Figure 1D).

### ACC Deaminase Producing Fungal Endophyte Screening Through *eto1* Bioassay

**FIGURE 2:**
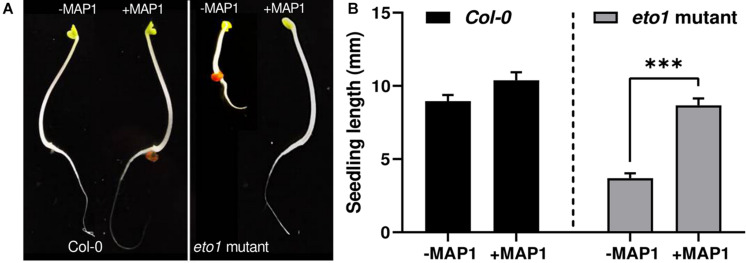
*eto1*-MAP1 co-culturing assay. **(A)** Phenotypic analysis showing comparison of MAP1-inoculated, etiolated seedlings of Col-0 accession and *eto1* mutant (Col-0 background) with non-inoculated seedlings. **(B)** Growth kinetics of Col-0 and *eto1* mutant seedlings. Quantitative data represent means ± *SD* of three independent experiments and at least 10 technical replicates each. The asterisks indicate a significant difference compared to untreated control (^***^p≤ 0.005).

To evaluate the growth-modulating and ET bio-reduction potential of MAP1, a bioassay was performed by using the etiolated seedlings of *Arabidopsis ET-overproducer* (*eto1*) mutant. The ET-induced morphological defects and triple response phenotype of etiolated *etol* seedlings were reverted to wild-type phenotype compared to the control plants upon MAP1 inoculation (Figure 2A). Etiolated hypocotyl growth kinetics were calculated for etiolated *eto1* seedlings with and without the application of MAP1 CF, exhibiting the reversal in overall length of hypocotyls same as wild-type Col-0 (Figure 2B).

### Molecular Identification of Endophyte by Phylogenetic Analysis

Identification of MAP1 was carried out by genomic DNA extracted from fungal mycelia. The closely related sequences extracted were aligned by using CLUSTAL W through MEGA7, software (Kumar et al., 2016). The closely related 18 S rDNA sequence of MAP1 isolate showed maximum sequence homology (61%) with *T. asperellum* (data not shown).

### Physiological Changes in MAP1*-*Mediated Wheat Plants Under WS

#### Growth Phenotype and Shoot Fresh Weight

**FIGURE 3:**
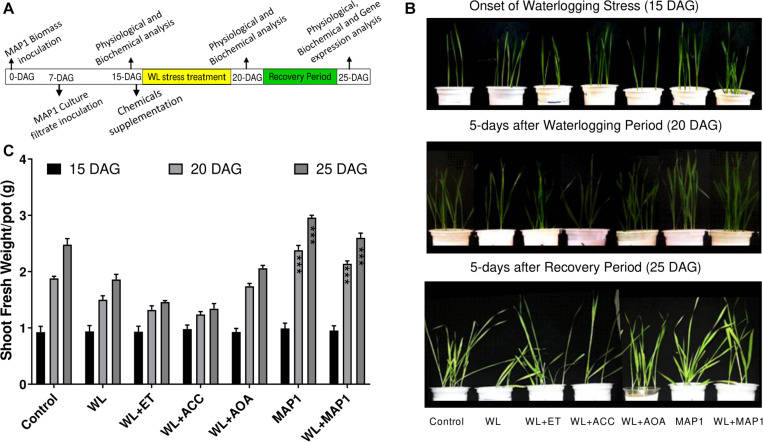
Effect of the fungal strain MAP1 on wheat plant growth. **(A)** Schematic representation of the wheat seedling bioassay experimental setup. Uniformly germinated wheat seedlings were transferred to the soil pots pre-mixed with MAP1 biomass. Seven-day-old wheat seedlings were inoculated with MAP1 culture filtrate. Fifteen-day-old seedlings were irrigated (8 h before onset of flooding treatment) with ACC, ethephon, and AOA. Fifteen-day-old plants were exposed to waterlogging stress for 5 days continuously and were subjected to a recovery period of further 5 days. Note the arrows indicating the time point of experimental activities. **(B)** Phenotypic analysis of wheat seedlings, with and without MAP1 inoculation exposed to WS and chemical supplementation. **(C)** Fresh weight of wheat seedlings compared to control. Quantitative data represent means ± *SD* of three independent experiments and at least 10 technical replicates each. The asterisks indicate a significant difference compared to untreated control (^***^p≤ 0.005).

Shoot fresh weight was increased in MAP1-inoculated wheat seedling under WS in comparison to non-inoculated seedlings, in accordance with the growth phenotype. Phenotypic analysis was performed at three different time points (Figure 3).

#### Effect of the Symbiotic Association on Host Plant’s Photosynthetic Activity, Growth Rate, and Stomatal Conductance

**FIGURE 4:**
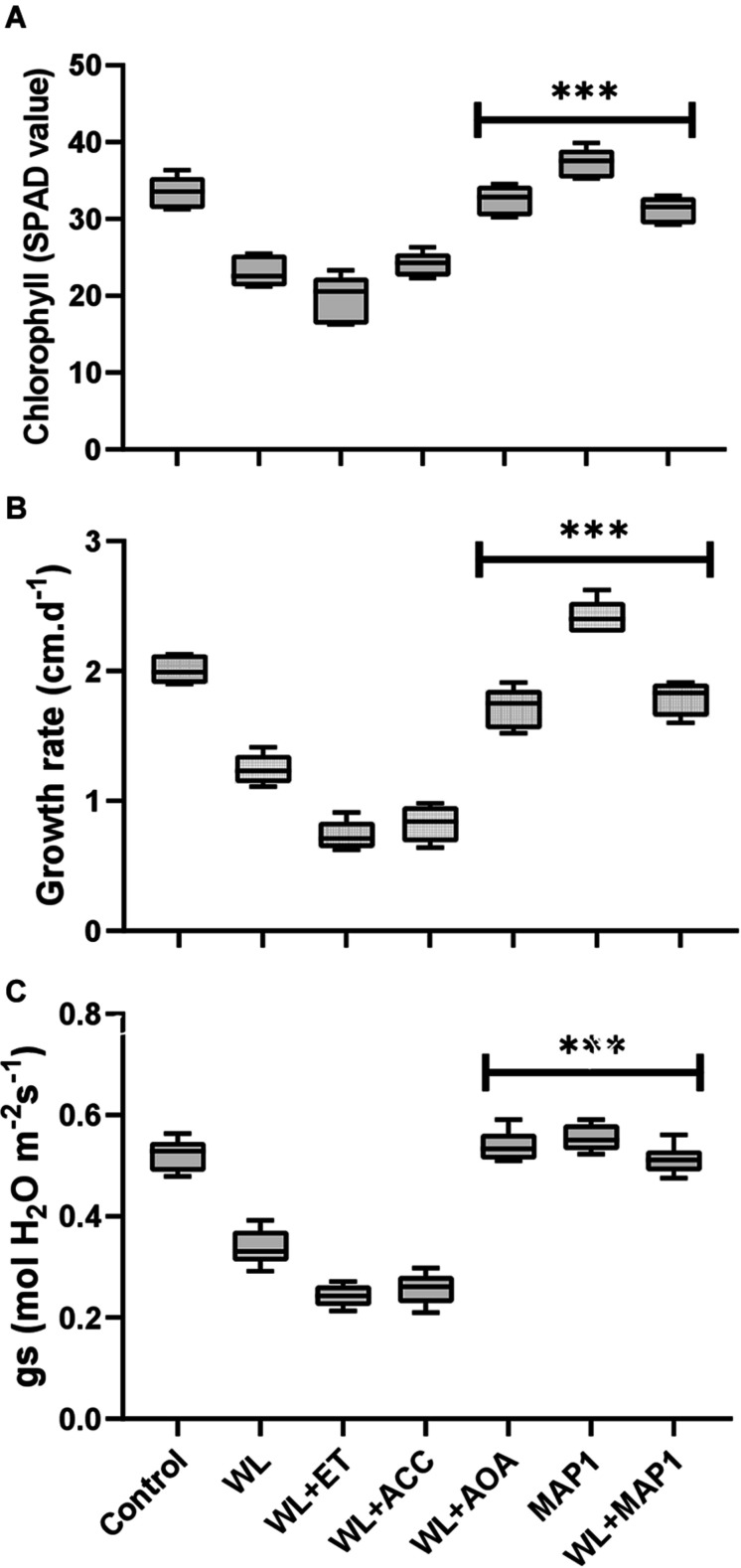
Determination of the effect of WS on **(A)** chlorophyll content, **(B)** growth rate, and **(C)** stomatal conductance of wheat plants with and without MAP1 inoculation. Quantitative data represent means ± *SD* of three independent experiments and at least six technical replicates each. The asterisks indicate a significant difference compared to untreated control (^***^p≤ 0.002).

The chlorophyll content, growth rate, and stomatal conductance are presented in Figure 4. A reduction in chlorophyll content, overall growth rate, and stomatal conductance was found in WS-treated plants. Further reduction was noticed by application of ACC and ethephon but reversed by AOA supplementation. MAP1 inoculation significantly increased the WS-tolerant of wheat plants compared with control, as indicated by modifications in chlorophyll content, overall growth rate, and stomatal conductance after WS treatment (Figure 4). A significant (*p* < 0.002) decrease in the chlorophyll content (35%), growth rate (40%), and stomatal conductance (35%) was recorded. Similarly, MAP1-inoculated plants showed significant (*p* < 0.002) increase in chlorophyll content (11%), growth rate (25%), and stomatal conductance (10%) in comparison to the non-inoculated seedlings (Figure 4).

#### Visualization and Quantification of Endogenous H_2_O_2_ Content in Wheat

**FIGURE 5:**
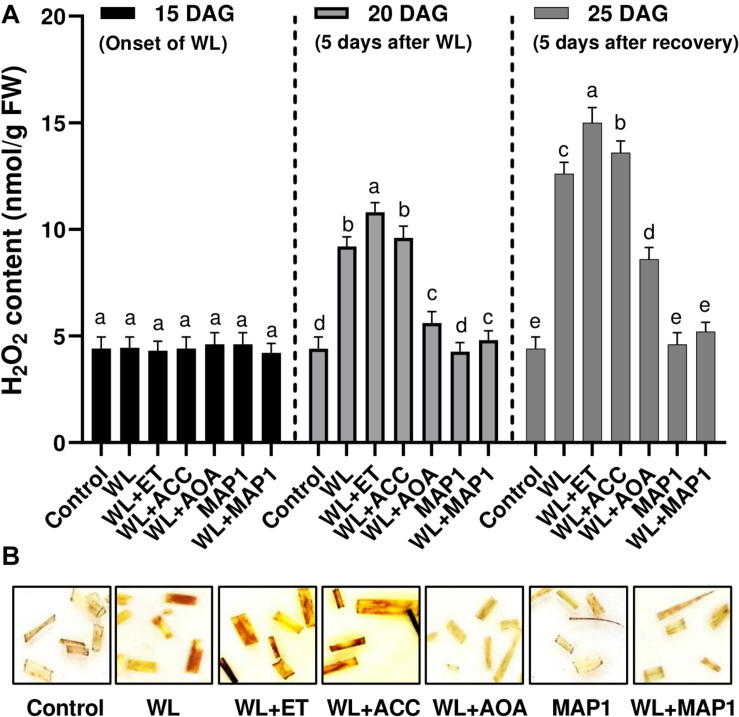
Effect of WS on **(A)** H_2_O_2_ production in wheat plants with and without MAP1 inoculation, exposed to WS. Quantitative data represent means ± *SD* of three independent experiments and at least 10 technical replicates each. **(B)** DAB staining using 1 cm-long segments of the 3rd leaf from 25-day-old seedlings with and without MAP1 inoculation. Different letters indicate statistically significant differences between treatments (*p* ≤ 0.05, DMRT).

Endogenous H_2_O_2_ content in 1-cm-long segments of the 3rd leaf from 25-day-old seedlings was visualized as brown precipitates through DAB staining procedure in Figure 5. Exposure of the wheat plants to the waterlogging application had a profound effect on the accumulation of reactive oxygen species in the form of H_2_O_2_, which was stronger upon supplementation of ethephon and ACC. Similarly, the supplementation of AOA and inoculation of MAP1 negatively affected the H_2_O_2_ production by lowering down the production of reactive oxygen species and mild brown staining in leaf segments in comparison to control (Figure 5).

#### MDA Content and Electrolyte Leakage in Wheat

**FIGURE 6:**
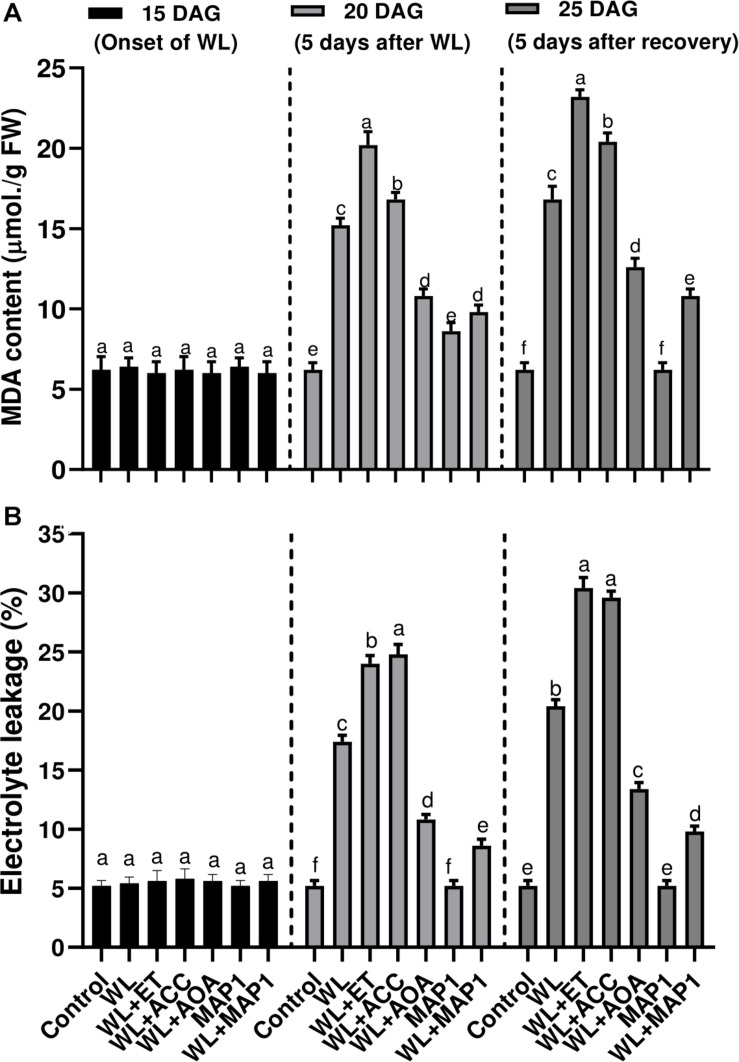
Effect of WS on **(A)** MDA content and **(B)** leaf electrolyte leakage in wheat plants with and without MAP1 inoculation, exposed to WS. Quantitative data represent means ± *SD* of three independent experiments and at least six technical replicates each. Different letters indicate statistically significant differences between treatments (*p* ≤ 0.002, DMRT).

Production of the MDA content and extent of the lipid peroxidation (LPO), in wheat plants under WS condition, was also evaluated. Results showed that WS led to an increase in LPO. Under WS, a higher MDA level was observed in non-inoculated seedlings (2.5-fold) compared with MAP1-inoculated plants (1.4-fold) (Figure 6A). Similarly, electrolyte leakage, due to LPO and biological membrane damages, was significantly (*p* < 0.002) increased in non-inoculated seedlings (3.4-fold) compared with MAP1-inoculated plants under WS (Figure 6B).

#### Antioxidant Enzyme Regulation in Wheat

**FIGURE 7:**
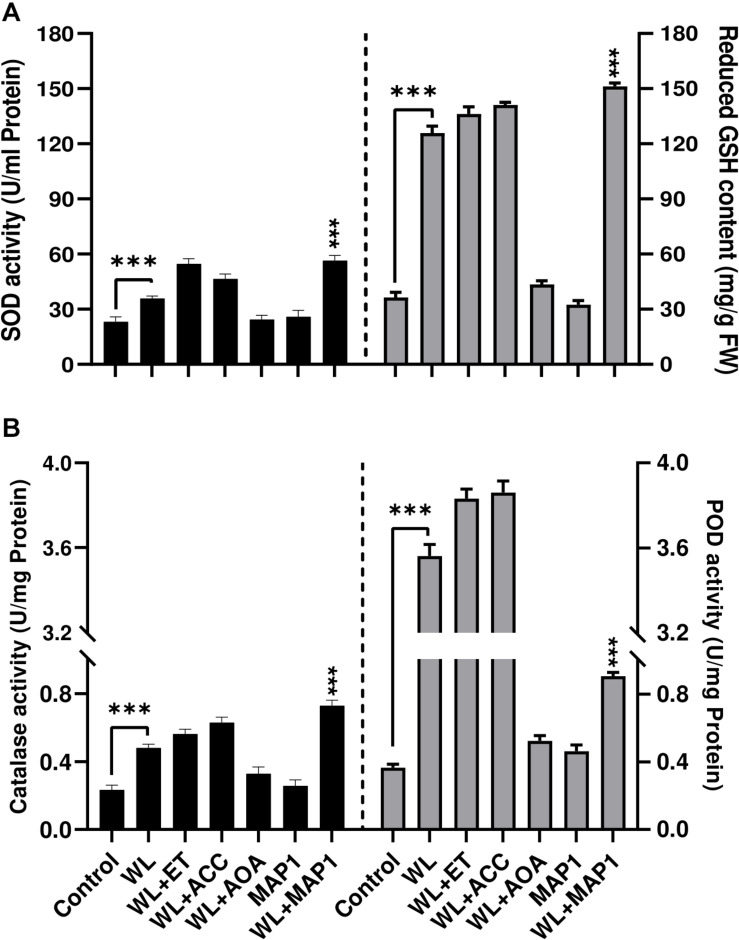
Effect of WS on antioxidant enzyme activity **(A)** SOD (left-axis), reduced glutathione (GSH) contents (right-axis) **(B)** CAT (left-axis), POD (right-axis) in wheat plants with and without MAP1 inoculation. Quantitative data represent means ± *SD* of three independent experiments and at least 10 technical replicates each. The asterisks indicate a significant difference compared to untreated control (^***^p≤ 0.005).

Antioxidant enzyme activities were also affected upon MAP1 inoculation in WS-exposed wheat plants. WS significantly (*p* < 0.002) induced superoxide dismutase (SOD) activity up to 1.8-fold and GSH up to 3.9-fold, in non-inoculated wheat plants, while MAP1 inoculation resulted in an increase in SOD activity up to 2.4-fold and glutathione (GSH) up to 5-fold (Figure 7A) as compared to control plants. Similarly, CAT and POD activity measurement revealed a significant (*p* < 0.05) increase in CAT activity up to 1.6-fold and POD up to 4-fold in non-inoculated wheat plants exposed to WS as compared to control plants. MAP1-inoculated plants showed a significant (*p* < 0.002) increase in CAT activity up to 3.5-fold and POD activity up to 4.5-fold, as compared to control plants (Figure 7B).

#### Effect of MAP1 on ACC Content and Polyamine Production in Wheat

**FIGURE 8:**
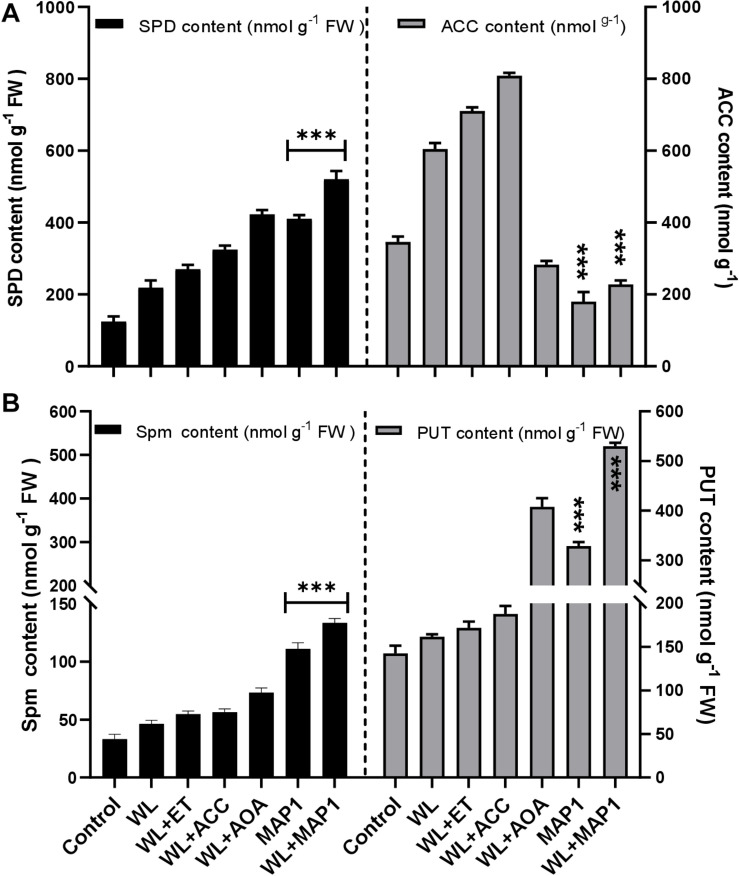
Effect of WS on free polyamines and ACC content. **(A)** Spermidine (left axis) and ACC content (right axis). **(B)** Spermine (left axis) and putrescine (right axis) in wheat plants with and without MAP1 inoculation. Quantitative data represent means ± *SD* of three independent experiments and at least 10 technical replicates each. The asterisks indicate a significant difference compared to untreated control (^***^p≤ 0.05).

ACC content was determined to analyze the regulation of the physiological function of the wheat plant under normal and WS condition with MAP1 inoculation as shown in Figure 8. The results revealed that there was significant (*p* < 0.05) increase in ACC content (71%) in MAP1 non-inoculated waterlogged wheat plants compared to control plants. However, MAP1 inoculation significantly (*p* < 0.05) lowered down the ACC content up to 54% as compared to control plants (Figure 8). Free polyamines were quantified and results showed that WS induced spermine, spermidine and putrescine in wheat plants. There was a significant (*p* < 0.002) increase in spermidine (2.1-fold), spermine (1.6-fold), and putrescine (1.2-fold) in non-inoculated waterlogged wheat plants in comparison to control. MAP1 inoculation further led to an elevated production of free polyamines upon WS. Results showed a significant (*p* < 0.002) increase in spermidine (5.7-fold), spermine (3.5-fold), and putrescine (3.4-fold) in MAP1-inoculated waterlogged wheat plants in comparison to control (Figure 8).

#### Effect of MAP1 on Gene Expression in Wheat

**FIGURE 9:**
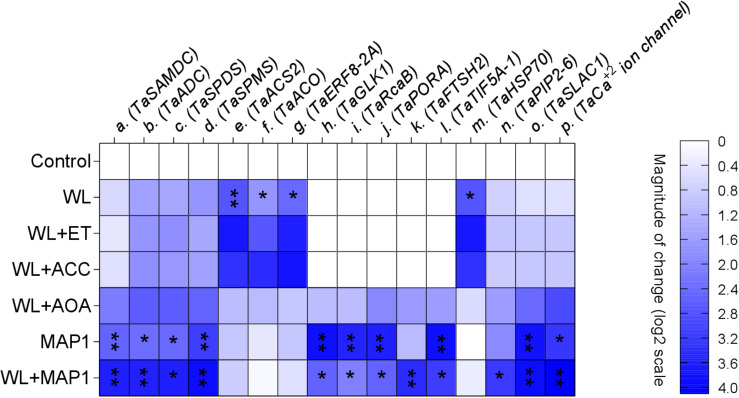
Expression profiling of polyamine biosynthesis genes, ET biosynthesis and signaling genes, photosynthetic activity-related genes, stress-reducing genes, and membrane transporter genes (root and stomatal) by qRT-PCR. Quantitative data represent means ± *SD* of three independent experiments and at least four technical replicates each. The asterisks indicate a significant change in gene expression compared to untreated control (^∗^*p*≤ 0.05; ^∗∗^*p*≤ 0.01).

The transcript abundance of polyamine biosynthesis, ET biosynthesis/signaling, cell wall expansion, root localized transporters, stomatal ion channels, and stress-related marker genes was evaluated, as shown in Figure 9. *S-adenosyl methionine decarboxylase* (*TaSAMDC*) expression was induced up to 1.5-fold, *arginine decarboxylase* (*TaADC*) 1.5-fold, *spermidine synthase* (*TaSPDS*) 1.4-fold, and *spermine synthase 2* (*TaSPMS*) 1.7-fold, in wheat plants exposed to WS in comparison to control plants. However, the further increase was noticed in the expression of *TaSAMDC* (3.4-fold), *TaADC* (3.3-fold), *TaSPDS* (3.5-fold), and *TaSPMS* (3.6-fold) in MAP1-inoculated waterlogged wheat plants as compared to control. Results showed that expression of *ET-responsive factor 8* (*ERF8-2A*) was induced up to 2.2-fold, *ACC oxidase* (*ACO*) 2.2-fold, and *ACC synthase 2* (*ACS2*) 2.4-fold, in wheat plants exposed to WS as compared to control plants (Figure 9). The expression of *ERF8-2A*, *ACO*, and *ACS2* was reduced up to 1-fold, in MAP1-inoculated waterlogged wheat plants as compared to control (Figure 9). Similarly, expression of *the golden 2-like protein* (*TaGLK1*) was induced up to 4.1-fold, *Ribulose bisphosphate carboxylase activase B* (*TaRcaB*) 4-fold, *Protochlorophyilide reductase* (*TaPORA*) 3.8-fold, and *ATP-dependent zinc metalloprotease* (*TaFTSH2*) and *Eukaryotic translation initiation factor 5A-1* (*TaTIF5A-1*) up to 4-fold in MAP1-inoculated seedlings compared to control, while expression of *TaGlk1*, *TaRcaB*, *TaPORA*, *TaFTSH2*, and *TaTIF5A-1* was induced up to 3. 1−, 2. 3−, 2. 6−, 3. 8−, and 3.5-fold, respectively, in MAP1-inoculated, waterlogged seedlings compared to non-inoculated waterlogged seedlings (Figure 9). *Heat Shock Protein 70* (*TaHSP70*) was induced in waterlogged wheat up to 3.1-fold, and reduced upon MAP1 inoculation up to 0.2-fold in comparison to control plants (Figure 9). *Aquaporin* (*TaPIP2-6*), *Guard cell S-type anion channel* (*TaSLAC1*), and *T. aestivum calcium ion channel* (*TaCa^+2^ ion channel*) were induced up to 2. 7−, 3. 6−, and 3.7-fold in MAP1-inoculated waterlogged wheat as compared to control, respectively (Figure 9).

## Discussion

Many strains of *Trichoderma* are known to produce ACC deaminase and IAA (Viterbo et al., 2010; Contreras-Cornejo et al., 2016). However, the role of ACC deaminase- and IAA-producing *Trichoderma* in various abiotic stresses, particularly WS tolerance, remains to be investigated in detail. To this end, the role of ACC deaminase-producing fungus (MAP1) was investigated in wheat seedlings under WS. In this study, MAP1 showed the capability to generate IAA, proline, total phenols, and flavonoid, while DPPH, free radical scavenging potential, and *ACCD* gene induction upon ACC supplementation have also been observed, indicating its potential role for induction of stress response and tolerance mechanism in plants. Endogenous phytohormone level also fluctuates due to environmental perturbance that modulates plant growth for better survival. IAA is one of the major phytohormones playing a crucial role in controlling the growth and development of plants. The previous study has demonstrated that exogenous IAA promoted wheat plant survival upon low oxygen and flooding by a reduction in electrolyte leakage and reactive species (singlet oxygen, hydroxyl radical, superoxide anion, and hydrogen peroxide), which cause damage to cellular organelles and induce toxicity and LPO (Yemelyanov et al., 2020). Additionally, IAA can increase the abiotic stress tolerance through the provocation of gene expression, antioxidant enzyme activity, photosynthetic pigment accumulation, and osmoprotectant (proline) synthesis that act as a free radical scavenger, thereby overcoming oxidative stress (Siddiqui et al., 2017). WS is known to increase ET levels in plants, which often causes a significant decrease in shoot and root growth, inducing leaf senescence and abscission (Najeeb et al., 2018) as well as a reduction in photosynthesis (Rajala and Peltonen-Sainio, 2001; Pierik et al., 2007). It is well known that many species of *Penicillium* and *Trichoderma* have ACC deaminase ability to cleave the ET precursor ACC to a-ketobutyrate and ammonia, due to possession of *ACCD* gene (Jia et al., 1999; Hontzeas et al., 2005; Yim et al., 2013). Two putative *ACCD* genes have been found in the genome of *A. thaliana*, and these genes act as regulators of ACC level in *A. thaliana* and also in tomato, during fruit development (McDonnell et al., 2009; Plett et al., 2009). Some species of *Trichoderma* harboring *ACCD* gene has shown beneficial effects on the plant growth and enhanced resistance to both biotic and abiotic stresses (Harman et al., 2004). To our knowledge, there is no previous study on ACC deaminase-producing fungal endophytes for growth promotion and WS alleviation through bio-reduction of ET in wheat. WS can influence the molecular and biochemical prospects of plant growth and lead to morphological and physiological irregularities in plants (Rauf et al., 2013b; Bashar et al., 2019). It is reported that various endophytic fungi can act as external factories of phytohormones, enzymes, and secondary metabolites and therefore have an impact on growth attributes of respective mutant such as *GA-deficient rice mutant* (*Waito-C rice*) (Sturz et al., 2000; Guo et al., 2015). Similarly, several phytohormone-insensitive or overproducer mutants of *A. thaliana* have also been used to investigate effects of plant growth-promoting endophytes, such as *Bacillus megaterium* (López-Bucio et al., 2007), *Phyllobacterium brassicacearum* (Galland et al., 2012) on root architecture, and ACC deaminase producing *Variovorax paradoxus* on *eto1* mutants (Chen et al., 2013). To evaluate the growth modulating and ET-reduction potential of deaminase-producing fungi *T. asperellum* (MAP1), *in planta* bioassay was performed by using the etiolated seedlings of *Arabidopsis ET-overproducer* (*eto1*) mutant. The ET-induced morphological defects and triple response phenotype of etiolated *etol* seedlings were reverted to wild-type phenotype compared to the control plants upon MAP1 inoculation. In the current study, WS tolerance negatively affected plant growth and development; however, inoculation with MAP1 isolate markedly alleviated the adverse effects of WS in wheat seedlings. It has long been known that soluble carbohydrates, proline, glycinebetain, and polyamines have a cryoprotective function. The environmental stresses like high temperature, drought, and waterlogging slow down crop growth and speed up the accumulation of biochemicals like proline that indicates metabolic re-adjustments for stress tolerance in wheat (Guy, 1990; Ahmed et al., 2017; Naeemi et al., 2018). In the current study, tolerance response was observed in MAP1-inoculated wheat plants exposed to WS. Necrosis, chlorosis, and reduced productivity are the main symptoms that appeared in plants exposed to prolonged waterlogging-induced hypoxic conditions (Paul et al., 2016). Our findings showed that when plants were exposed to WS, a decline in the chlorophyll, growth rate, and stomatal conductance was observed; however, MAP1 inoculation modulated WS response by increasing chlorophyll content, growth rate, and stomatal conductance. Besides, reactive oxygen species (H_2_O_2_) production was increased in WS-exposed wheat plants, but MAP1 inoculation contributed to a decrease in H_2_O_2_ production. It can be co-related with previously known ACC deaminase-producing endophytes such that a decrease in ACC and ET content roughly up to 2- to 4-fold contributes to a reversal of the adverse effects of WS on plants (Grichko and Glick, 2001; Mayak et al., 2004). However, plants initiate a complicated antioxidant defense system composed of glutathione reductase (GR), SOD, and APX, which rescue the plants against cellular stress, deplete free radicals, and scavenge excessive ROS. Compared with non-inoculated wheat plants under WS, MAP1-inoculated plants exhibited less electrolyte leakage and MDA content due to a reduction in LPO of the cellular membrane. Higher SOD, CAT, and POD activity and higher reduced GHS content were also observed in MAP1-inoculated wheat plants. Inoculation of MAP1 after WS enables wheat plants to minimize ROS by enhancing the antioxidant activity. A similar observation was noticed by Xue et al. (2020), who revealed that mild flooding quickly compels plants to produce ROS at the appropriate level, while severe flooding results in ROS over-accumulation exceeding scavenging activity, adversely affecting the plant growth and yield. Amino acids play an important role in the biochemical and physiological mechanisms of plants by modifying membrane permeability, regulating the transport of osmolytes and ions for enhancing abiotic stress tolerance. Polyamines such as spermidine (Spd), spermine (Spm), and putrescine (Put) are low-molecular-weight, aliphatic nitrogenous compounds (Groppa and Benavides, 2008) known as plant growth regulators (Tavladoraki et al., 2012). Polyamine metabolism is associated with the response of plant’s response toward changing environmental conditions, such as high temperature, drought, salinity, flooding, and nutrient deficiency (Zhao et al., 2017), due to their role in protecting membrane, stabilizing nucleic acids and protein structures, and scavenging free radicals (Alcázar et al., 2010). Biosynthesis and further catabolism of polyamines are interconnected with other metabolic pathways that function in plant stress tolerance (Alcázar et al., 2010). The interconnectivity between the stress-induced polyamines and ET metabolism reflects that their biosynthetic pathways share SAM as a common substrate, but their functions are diametric (Apelbaum et al., 1981). For evaluating the interconnection between the stress-inducible polyamines and ET metabolic pathway, the free polyamines (spermine, spermidine, and putrescine) and ACC contents were quantified showing higher free polyamine content and lower ACC content, in MAP1-inoculated waterlogged wheat plants as compared with non-inoculated seedlings, indicating the ability of ACC deaminase-producing MAP1 isolate to catalyze the endogenous ACC content of wheat, for controlled ET production during WS. Thus, MAP1 exerted its rescuing effect through interconversion of polyamines and ET metabolism during the waterlogged condition in wheat plants. The ultimate rescuing response of a plant to WS is the upregulation of stress-related genes for synthesis of stress-relieving metabolites (Rauf et al., 2013b; Voesenek and Sasidharan, 2013). Previously, group VII ET response factors (*ERFVIIs*) have been reported to play important roles in ET signaling and plant responses to flooding. For example, *ZmEREB180* was tightly associated with WT and its expression was up−regulated by ET (Yu et al., 2019). Previously, transcriptomic changes have been performed in wheat roots inoculated by *T. harzianum*, supplemented with nitrogen (N) source revealing the role of *Trichoderma* as a plant defense inducer (Rubio et al., 2019). In the present study, gene expression analysis revealed the higher expression of ET biosynthesis/signaling genes such as *ERF8-2A*, *ACO*, and *ACS2*, upon WS alone and in combination with ethephone and ACC treatment. In contrast, the reduced expression of ET biosynthesis/signaling genes was observed in MAP1-inoculated wheat plants exposed to WS compared to non-inoculated seedlings. Moreover, the expression related to free polyamines biosynthesis genes (*TaSAMDC*, *TaADC*, *TaSPDS*, and *TaSPMS*) was upregulated in MAP1-inoculated wheat plants exposed to WS compared to non-inoculated seedlings (Figure 9). This observation exposed not only the reduction of ET due to deaminase activity by MAP1 but also its role in cell signaling mechanism triggering the gene expression in plants. This underlying unknown molecular mechanism for microbe-plant cell signaling is yet to be explored. Similarly, the expression of *TaGlk1* involved in the maintenance of photosynthetic apparatus, *TaPORA* related to chlorophyll biosynthesis (Rauf et al., 2013a), *TaRcaB* gene for fixation of CO_2_ (Saeed et al., 2016), *TaFTSH2* gene for photosystem II repair, and *TaTIF5A-1* gene for protein biosynthesis was downregulated in waterlogging stressed wheat plants, but differentially induced in MAP1-inoculated, waterlogged stressed plants compared to non-inoculated waterlogged wheat. Heat shock proteins (HSPs) are major “molecular chaperones” that act as protein folding catalysts and may enhance tolerance against stresses. Previously, 20 HSPs have been documented to be highly accumulated due to low oxygen stress on wheat plants, and *HSP70* was the most abundant among them (Pan et al., 2019). Similarly, in the current study, *HSP70* transcript abundance was found to be higher in non-inoculated waterlogged seedlings compared to MAP1-inoculated waterlogged seedlings, suggesting that high abundance of HSP70 is required in preventing aggregation of the denatured and misfolded proteins under WS. Likewise, the expression of the *TaPIP2-6* gene for water channel proteins “aquaporin” (AQP), *TaSLAC1* gene for guard cell S-type anion channel, and *TaCa^+2^ ion channel* (*T. asetivum* calcium ion channel) was differentially induced in MAP1-inoculated, waterlogged stressed plants compared to non-inoculated waterlogged wheat (Figure 9). Moreover, it is known that ET induces CuAO (copper amine oxidase) and oxidation of polyamines like putrescine by CuAO produces H_2_O_2_ and follows stomatal closure in *Vicia faba* (Song et al., 2014). These findings consistently suggest that *TaSLAC1*, *TaPIP2-6*, and *TaCa^+2^ ion channel* may also be involved in plant WS tolerance by regulating the optimal transport of K^+^ and of Ca^+2^ to maintain water potential in stomatal guard cells for controlling the stomatal closure and transpirational activities for plant survival, better growth, and yield, under WS.

**FIGURE 10:**
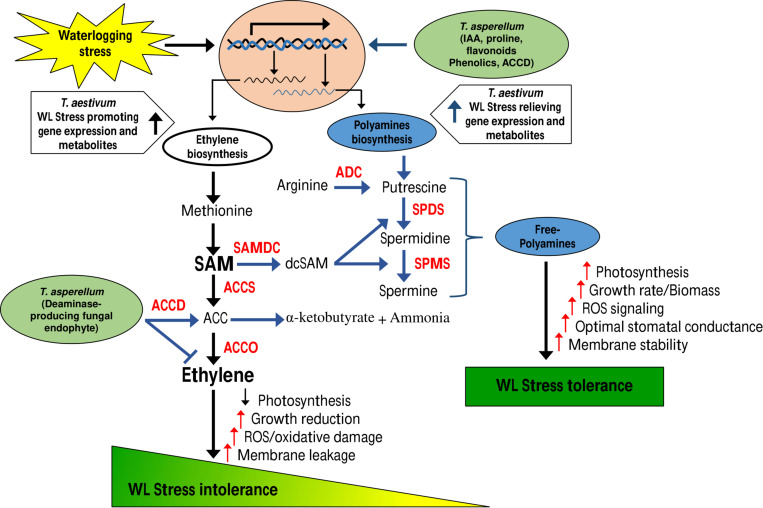
Model of *T. asperellum* MAP1 action during waterlogging-induced stress.

Collectively, our data suggest a regulatory model (Figure 10) whereby *T. asperellum* MAP1 isolate plays a decisive role in alleviating WS and promoting growth; MAP1 is exhibiting two major modes of actions. (i) MAP1 is directly reducing ET overproduction via TasACCD enzymatic activity on ACC content of wheat plants under WS. (ii) MAP1 indirectly modulates the expression of ET biosynthesis/signaling genes and polyamine biosynthesis genes, by inducing the expression of unknown signaling factors. Overproduced ET leads to reduced photosynthesis/growth rate and increased ROS/oxidative damage/membrane leakage, in wheat plants without MAP1 inoculation. ACC deaminase-producing fungal endophyte *T. asperellum* MAP1 isolate reduces the overproduced ET by deaminase activity and converts ACC to α-ketobutyrate and ammonia. ACCD-producing MAP1 induced the expression of polyamine biosynthesis genes further modulating the expression of photosynthetic activity-related genes, stress-reducing genes, and membrane transporter genes (root and stomatal), through unknown signaling networks. MAP1 inoculation facilitates better photosynthetic activity, promotes growth rate/biomass, induces ROS signaling, optimizes stomatal conductance, and stabilizes cellular membrane in wheat plants exposed to WS, hence alleviating WS and promoting growth.

## Conclusion

In conclusion, the present study revealed the potential of *T. asperellum* MAP1 to produce IAA, phenols, and flavonoids. MAP1 inoculation enhanced overall wheat growth in terms of higher chlorophyll content, accelerated growth rate, and increased stomatal conductance leading to higher biomass under normal as well as WS. This improved plant growth was coupled with the increased content of endogenous free polyamine (spermine, spermidine, and putrescine) and decreased ACC content (precursor of ET). Gene expression modulation was also governed by MAP1 for polyamine biosynthesis and ET biosynthesis/signaling genes. Active plant growth-promoting strain MAP1 is suitable for the production of biofertilizers for growth promotion and quantified for WS tolerance. Moreover, further investigation with a focus on fungal–plant signaling mechanism is needed to understand the underlying molecular base in WS tolerance in wheat plants.

## Data Availability Statement

Dataset for sequencing of the Internal Transcribed Spacer (ITS) of 18S rDNA for MAP1, performed in the present study, can be found in the online repository. https://www.ncbi.nlm.nih.gov/genbank/, MW015788.1.

## Author Contributions

MR and MAr initiated, designed, and performed the main experiments, and wrote the manuscript. MAw, AU-D, KA, HG, MR, and MH contributed to the research work and approved the submitted version of this article. All authors contributed to the article and approved the submitted version.

## Conflict of Interest

The authors declare that the research was conducted in the absence of any commercial or financial relationships that could be construed as a potential conflict of interest.
